# Boosting brain glucose metabolism to fight neurodegeneration?

**DOI:** 10.18632/oncotarget.15131

**Published:** 2017-02-07

**Authors:** María L. de Ceballos, Attila Köfalvi

**Affiliations:** Department of Translational Neuroscience and Centre for Biomedical Networked Research on Neurodegenerative Diseases, Neurodegeneration Group, Spain, Cajal Institute, CSIC, Madrid, Spain

**Keywords:** Alzheimer's disease, cannabinoid CB2 receptor, ^18^FDG-PET, hypometabolism

Alzheimer's disease (AD) is the main cause of dementia in the elderly population with increasing prevalence. Despite the huge efforts invested in AD research, no therapeutic breakthrough has been witnessed yet. Likely, novel approaches and sweeping ideas will be necessary to effectively treat AD. Nevertheless, the earlier the disease is detected the more efficacious the therapy is. Early detection in presymptomatic patients at risk encompasses psychological tests, *in vivo* amyloid assessment and the evaluation of cerebral ^18^F-fluoro-2-deoxy-D-glucose uptake with the help of positron emission tomography (^18^FDG-PET). As most neuropsychiatric disorders, AD also has its own distinctive fingerprint of regional cerebral glucose dysmetabolism. In AD patients, lower ^18^FDG-PET signals are found in the temporoparietal and posterior cingulate regions [1]. Since these metabolic alterations precede the first clinical symptoms and neurodegeneration by years, significant loss of neurons should not be accounted for the lower ^18^FDG-PET signal. As glucose is the predominant source of energy for the brain cells, lower uptake rates may represent a reduced energy demand in the affected area - a passive consequence of the illness.

However, in our opinion, either difficulties in glucose transport from the circulation to the brain cells or inefficient glucose metabolism or both can also generate reduced ^18^FDG-PET signal. We are firmly convinced that under any of these conditions, neurons would be energy deprived in times when they need to spend more energy than normally, on dealing with *e.g.* misfolded proteins, Ca^2+^ deregulation or synaptic integrity. According to this hypothesis, regional hypometabolism may actively facilitate disease progression. In other words, cerebral regional hypometabolism would qualify as a druggable target, beyond being of precious diagnostic value. Indeed, AD is also termed as cerebral diabetes, and cerebral insulin resistance is one major etiological factor of sporadic AD [2].

Which are the concrete drug targets to boost brain glucose use? It is common-sense to look for them among those neuromodulator systems that become activated under synaptic firing, such as endocannabinoids. The endocannabinoid system in *sensu stricto* comprises the most studied receptors and endogenous ligands, *i.e.* the G protein-coupled cannabinoid CB_1_ and CB_2_ receptors (CB_1_R and CB_2_R), and the arachidonate-derivative endocannabinoids, anandamide and 2-AG, along with their synthetic and degradation enzymes including cyclooxygenase-2 [4]. The peripheral glucoregulator role of the endocannabinoid system is well-known, and the CB_1_R was targeted with the later redrawn antiobesity medicine, Acomplia [4]. In the brain, endocannabinoids are released primarily upon converging excitatory and/or neuromodulator inputs to reduce the activity of synapses equipped with the CB_1_R [4]. While the CB_1_R is responsible for the psychoactivity of marijuana's Δ^9^-THC, the cerebral role of the non-psychoactive CB_2_R remains much less understood. Novel data suggest that this receptor is expressed by neurons in the healthy brain, but in AD, CB_2_R expression will be predominant in activated microglia, that wreak havoc on neurons [4]. Recently, numerous novel CB_2_R-selective agonists have been created and investigated even in clinical trials in conditions involving central and peripheral neuropathy, since CB_2_R activation is antiinflammatory and analgesic [4,5]. Indeed, we and others have reported previously that CB_2_R activation ameliorates or normalizes major hallmarks of AD, such as cognitive deficits, neuroinflammation, glial activation and β-amyloidosis [6].

This time, we asked if endocannabinoids via CB_2_R activation could link increased neural activity with greater energy intake. This study was carried out in mice, and we found that CB_2_R activation stimulates glucose uptake on the minute scale in brain regions corresponding to those typically affected by AD in humans [3]. First, we observed the metabolic booster effects of CB_2_R agonists in two different *in vitro* models - astrocytic and neuronal cultures and acute brain slices of young mice-, employing either fluorescently or radioactively labeled glucose analogues. Nonetheless, the onset of sporadic AD mostly affects middle-aged and elderly people. Therefore, it was essential that we could repeat the above findings in middle-aged mice. These mice served as control for the middle-aged transgenic β-amyloidosis model TgAPP 2576 mice, which expresses the double-mutant human amyloid precursor protein, and thus, in good part recapitulates human AD. Interestingly, exogenous CB_2_R activation still stimulated glucose uptake in the TgAPP 2576 brain slices, but boosting endocannabinoid levels via the blockade of cyclooxygenase-2 stimulated glucose uptake only in the control mice. With additional assays we established that it is likely anandamide rather than 2-AG that stimulates glucose uptake via CB_2_R. We also found that β-amyloidosis reduced hippocampal anandamide levels by 30%, which should explain why cyclooxygenase-2 blockade failed to stimulate glucose uptake in the TgAPP 2576: there were simply not enough basal anandamide levels to boost. Importantly, we could repeat our findings *in vivo* with the help of micro ^18^FDG-PET, and we saw that CB_2_R agonists stimulate glucose uptake up to 130% of baseline in the brain of both the control and the human AD mice model.

These data identifies the CB_2_R as a druggable target because CB_2_R agonists can stimulate cerebral glucose uptake both in the young and the elderly populations, and that endogenous CB_2_R activation might be hampered by β-amyloid and cyclooxygenase-2, leading to hypometabolism in AD. The emphasis is put on “CB_2_R-selectivity”. Δ^9^-THC activates the CB_1_R, too, thus marijuana does not seem to qualify for an AD therapy for several reasons: 1) The activation of CB_1_Rs lowers mitochondrial metabolism of glucose in the hippocampus [7]; 2) CB_1_R activation by pathologically increased 2-AG signalling may contribute to synaptic deficits in human AD [8], and 3) CB_1_R activation impairs insulin signalling, even in the brain [4].
